# Characteristic radiological findings for revision surgery after balloon kyphoplasty

**DOI:** 10.1038/s41598-019-55054-5

**Published:** 2019-12-06

**Authors:** Shinji Takahashi, Masatoshi Hoshino, Hiroyuki Yasuda, Yusuke Hori, Shoichiro Ohyama, Hidetomi Terai, Kazunori Hayashi, Tadao Tsujio, Hiroshi Kono, Akinobu Suzuki, Koji Tamai, Hiromitsu Toyoda, Sho Dohzono, Ryuichi Sasaoka, Fumiaki Kanematsu, Masaki Terakawa, Hiroaki Nakamura

**Affiliations:** 1 Department of Orthopaedic Surgery, Osaka City University Graduate School of Medicine, Osaka, Japan; 2 Department of Orthopaedic Surgery, Osaka General Hospital of West Japan Railway Company, Osaka, Japan; 3 Department of Orthopaedic Surgery, Shimada Hospital, Osaka, Japan; 4 Department of Orthopaedic Surgery, Shiraniwa Hospital, Nara, Japan; 5 Department of Orthopaedic Surgery, Ishikiri Seiki Hospital, Osaka, Japan; 6 Department of Orthopaedic Surgery, Yodogawa Christian Hospital, Osaka, Japan; 7 Department of Orthopaedic Surgery, Saiseikai Nakatsu Hospital, Osaka, Japan

**Keywords:** Risk factors, Medical research

## Abstract

Balloon kyphoplasty (BKP) sometimes fails to improve patients’ outcomes, with revision surgery, using anterior or posterior reconstruction, being required. The purpose of this study was to investigate the radiological risk factors of failure after BKP in the treatment of osteoporotic vertebral fractures (OVFs). This case-control study included 105 patients treated with single BKP and 14 patients  who required revision BKP. We evaluated radiological findings differentiating both groups, using plain radiography and computed tomography, before BKP. Angular flexion-extension motion was significantly greater in the revision than BKP group. While the frequency of pedicle fracture and posterior wall injury was not different between the groups, a split type fracture was more frequent in the revision group. Split type fracture had the highest adjusted odds ratio (OR) for revision (16.5, p = 0.018). Angular motion ≥14° increased the risk for revision surgery by 6-fold (p = 0.013), with endplate deficit having an OR of revision of 5.0 (p = 0.032). The revision rate after BKP was 3.8%, with split type fracture, greater angular motion and large endplate deficit being risk factors for revision. Treatment strategies for patients with these risk factors should be carefully evaluated, considering the inherent difficulties in performing revision surgery after BKP.

## Introduction

Balloon kyphoplasty (BKP) is commonly performed in patients with osteoporotic vertebral fractures (OVFs)^1^, with its efficacy and safety having previously been reported. In particular, due to its low invasiveness, the procedure is effective for elderly patients. BKP can also improve the timeliness of the management of spinal fractures, which is critical to avoid secondary medical complications due to limited mobility associated with improperly treated fractures. Major complications of BKP occur in <1% of patients treated for OVFs^2^. However, several potential complications may occur, including extrusion of the cement into the spinal canal, with subsequent spinal cord injury, infection, hematoma formation, pulmonary embolus, failure to relieve pain, osteomyelitis, and adjacent vertebral fractures (AVFs)^3^. Moreover, the procedure sometimes fails to improve patients’ outcomes, requiring revision surgery, using anterior or posterior reconstruction, with revision surgery increasing the risk of infection^4^. As well, BKP is not indicated for vertebral fractures that include posterior wall injury, which is a common occurrence in OVFs^5^. Injury to the posterior wall in OVFs results from the fragility of the vertebra, a clearly different finding from spinal fractures in patients without osteoporosis. As involvement of the posterior wall is a relative contraindication for BKP, only a few studies have focused on this problem explicitly. One study reported BKP to be an effective procedure, with few complications, for the treatment of OVFs with partial inclusion of the posterior wall in elderly patients^5^. However, if BKP fails, revision surgery is necessary and is associated with various complications.

Cement leakage is a well-known complication of revision surgery, requiring emergency decompression for neurological complications. Delayed sequelae have also been reported, including cement leakage, AVFs, cement dislodgement or fragmentation, and pyogenic spondylitis^4,6–8^. However, there has been no reports to reveal the preoperative radiological risk factors of failure after BKP. Therefore, the purpose of our study was to investigate the preoperative radiological characteristics of failure after BKP.

## Materials and Methods

### Patients

**Figure 1:**
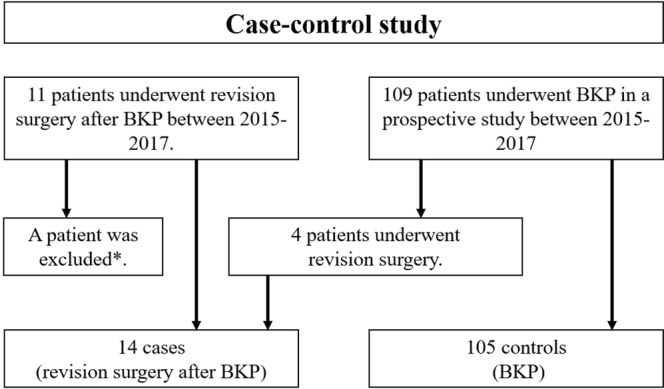
Flow chart for the study design. *A patient was excluded because infection was identified as the cause of revision surgery.

This was a case-control study including 14 patients who underwent revision surgery after BKP and 105 consecutive patients who underwent single BKP, serving as the control group (Fig. 1). Eligible patients were the 109 consecutive patients who underwent BKP in 11 of our affiliated institutions, from 2015 to 2017; the detailed methodology has been described previously^9,10^. Of these eligible patients, 4 patients required revision surgery. In addition, 11 patients were referred to the participating institutions for revision surgery in the same period, with one of these patients excluded as infection was the cause of revision surgery. Inclusion criteria for BKP were OVFs with instability between the flexion and extension position, viewed on lateral radiographs, a visual analog scale (VAS) pain score ≥4 and decreased bone density (T scores – 1). In addition, high intensity (similar to cerebrospinal fluid) or diffuse low intensity areas on T2-weighted magnetic resonance imaging (MRI) within 1 month after pain onset was an inclusion criterion. The MRI findings were associated with delayed union and residual intractable back pain^11^. Exclusion criteria were pathological fractures, suspected underlying malignant disease, infection as the cause of revision, and dementia. Study eligibility was determined after initial clinical and radiographic evaluation. All 109 consecutive patients included in our analysis were followed-up for at least 6 months after surgery.

Each patient underwent plain radiography and computed tomography (CT) of the thoracic and lumbar spine. Apart from surgical management, all patients received appropriate medical support, including non-steroidal anti-inflammatory drugs, and osteoporosis treatment, including daily or weekly teriparatide and other drugs (bisphosphonate, denosumab, and selective estrogen modulators). Participants provided written informed consent before enrollment.

### Balloon kyphoplasty

BKP was performed using the Kyphon Inc system (Medtronic Spine LLC, Sunnyvale, CA, USA), under general anesthesia^12^. Patients were positioned in prone, on a four-poster frame, without an excessive reduction of the kyphosis. A deflated balloon was inserted into the vertebral body, using a bilateral transpedicular approach, and inflated to restore the collapsed vertebral body to its normal height position, and to create an internal cavity under manometric control (with a maximum of 200 psi). The balloon was then deflated and withdrawn. The remaining cavity was filled, under low pressure, with polymethylmethacrylate (PMMA), to two-thirds of the distance from the anterior to the posterior vertebral cortex observed on a lateral fluoroscopic view. After surgery, patients with lumbar spine fractures were instructed to wear a tailor-made corset when out of bed. Physical therapy was started on postoperative day 1 to facilitate ambulation. All patients received analgesics, physiotherapy, rehabilitation, and walking aids, as needed, according to the standard practices of participating physicians and hospitals. Braces were prescribed for 98% of the patients (tailor-made hard braces, 38%; tailor-made elastic braces, 57%; ready-made elastic braces, 3%; and no brace, 2%).

### Revision surgery

Revision surgery was performed for uncontrolled back pain or neurological deficit after BKP, due to cement dislodgement, recollapse of the vertebra, and adjacent vertebral fracture. The clinical reasons for revision surgery included back pain in 13 patients and leg pain in 1 patient (Table 1). Posterior fusion was performed in 10 patients. Anterior and posterior fusion were performed in 4 patients for cement dislodgement repair. During posterior fusion, posterior instrumentation and bone grafting were performed, without cement removal. In anterior and posterior fusion, cement removal and bone grafting were performed, using an anterior approach, and posterior instrumentation was added.

**Table 1:** Characteristics of each patient who underwent revision surgery after BKP.

	Age	Sex	Index level	Risk	Surgery	Clinical reason	Cement migration
1	83	Male	L1	Split type	Posterior	Back pain	Anterior
2	74	Female	L2	Angular motion & endplate deficit	Posterior and Anterior	Back pain	Anterior
3	81	Female	L2	Angular motion & endplate deficit	Posterior	Back pain	Anterior
4	78	Female	T12	Angular motion & endplate deficit	Posterior	Back pain	Inferior
5	80	Male	T12	Angular motion	Posterior	Back pain	Anterior
6	83	Male	T12	Split type	Posterior	Back pain	Anterior
7	79	Male	T11	Angular motion	Posterior	Back pain	Inferior
8	76	Female	L4	Split type	Posterior & Anterior	Leg pain	Inferior
9	85	Male	T12	Endplate deficit	Posterior	Back pain	Superior
10	68	Male	L3	Angular motion	Posterior	Back pain	Anterior
11	85	Female	L3	Angular motion	Posterior	Back pain	Inferior
12	87	Male	L1	Endplate deficit	Posterior	Back pain	Superior
13	74	Female	L1	Angular motion & endplate deficit	Posterior & Anterior	Back pain	Anterior
14	83	Male	T12	Angular motion & endplate deficit	Posterior & Anterior	Back pain	Superior

BKP, balloon kyphoplasty.

### Image assessment

The type of fracture was classified using the AO classification, based on CT images as previously described^13^. However, since no commonly accepted classification for OVFs exists, we evaluated the characteristics of each fracture from lateral view plain radiographs, between the position of flexion and extension, and CT images, obtained before surgery. The wedge angle of the collapsed vertebral bodies was measured before surgery and at 1-week after surgery. The presence of pedicle and spinous process fracture was verified. Diffuse idiopathic skeletal hyperostosis (DISH) was diagnosed based on the presence of ossification observed along the anterolateral aspect of at least four contiguous vertebrae. Endplate deficit was defined as a >3 mm deficit in the upper or lower endplate.

### Statistical analysis

The chi-squared (χ^2^) test or Fisher’s exact test was used for categorical variables, whereas the *t*-test was used for continuous variables. The receiver operating characteristic (ROC) curve was used to investigate the area under curve (AUC) of the vertebral angular motion between the positions of flexion and extension, for revision surgery. The odds ratio (OR) of each pre-operative variable for revision surgery was calculated using a logistic regression model. The potential confounding factors were included in the multivariate analysis as follows: age, sex, angular motion ≥14°, DISH, endplate deficit, and split type fracture. Statistical test results were considered significant at p < 0.05. All p-values were two-sided. All analyses were performed using SAS version 9.4 software (SAS Institute, Cary, NC).

### Ethical approval

This study protocol was approved by the Institutional Review Board of Osaka City university (No. 3174). All methods were performed in accordance with the Declaration of Helsinki and the Ethical Guidelines for Medical and Health Research Involving Human Subjects in Japan.

## Results

The baseline characteristics between the revision and BKP groups are reported in Table 2. There was no difference in age between the two groups. The revision group had a higher proportion of males and significantly greater angular motion between the positions of flexion and extension (15.5° *versus* 8.6°, p < 0.001). The wedge angle of the vertebral body was similar between the two groups before BKP (21.7° *versus* 17.6°, p = 0.116), but was significantly smaller in the revision group after surgery (5.2° *versus* 12.2°, p < 0.001). Although DISH was more frequent in the revision group, this difference was not significant (p = 0.115). The frequency of spinous process fracture, pedicle fracture, and posterior wall injury were not different between the two groups; however, a split type fracture and endplate deficit (>3 mm) were more frequent in the revision group (21% *versus* 2%, p = 0.012 and 43% *versus* 10%, p = 0.004).

**Table 2:** Difference in baseline characteristics between patients who underwent balloon kyphoplasty only and those who required revision surgery after balloon kyphoplasty.

	Revision	BKP	P-value
	n = 14	n = 105	
	Mean (SD) or N (%)	Mean (SD) or N (%)	
Age, years	79.7(5.3)	79.2 (5.5)	0.737
Sex, male	8 (57%)	21 (20%)	0.005
**Affected level**			
Thoracic level (T7-T10)	0	3 (3%)	0.594
Thoraco-lumbar level (T11-L2)	11 (79%)	89 (85%)	
Lumbar level (L3-L4)	3 (21%)	13 (12%)	
**AO classification**			
A1	1 (7%)	22 (21%)	0.177
A2	0	0	
A3	5 (36%)	17 (16%)	
A4	8 (57%)	66 (63%)	
Angular motion between flexion and extension position preop, degrees	15.5 (7.1)	8.6 (5.9)	<0.001
Wedged angle preop, degrees	21.7 (8.3)	17.6 (7.2)	0.116
Wedged angle postop, degrees	5.2 (5.0)	12.2 (6)	< 0.001
Reduction, degrees	16.5 (8.2)	5.7 (6.2)	< 0.001
Old OVF	7 (50%)	50 (48%)	0.867
DISH	4 (29%)	13 (12%)	0.115
Split type fracture	3 (21%)	2 (2%)	0.012
Spinous process fracture	3 (21%)	14 (13%)	0.420
Pedicle fracture	3 (21%)	14 (13%)	0.420
Endplate deficit (>3 mm)	6 (43%)	10 (10%)	0.004
Posterior wall injury	13 (93%)	83 (79%)	0.299

BKP, balloon kyphoplasty; preop, pre-operatively; postop, postoperatively; DISH, diffuse idiopathic skeletal hyperostosis; OVF, osteoporotic vertebral fracture.

**Figure 2:**
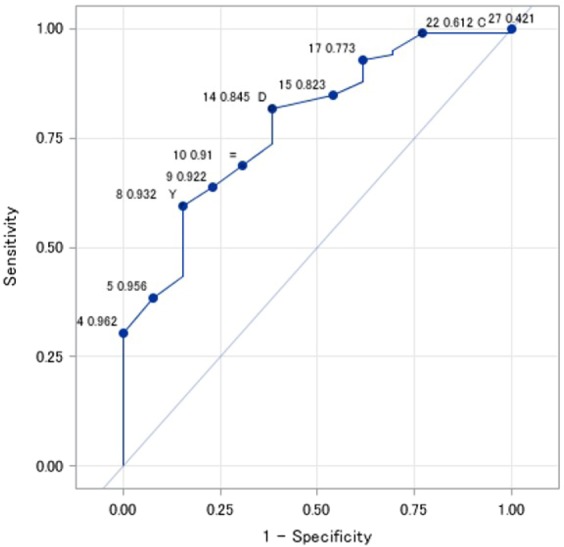
Cutoff value of vertebral angular motion before balloon kyphoplasty. The receiver operating characteristic (ROC) curve was used to investigate the relationship between vertebral angular motion and re-operation. The distance from the top left corner of the ROC curve was used to determine the cutoff value of vertebral angular motion for re-operation, with an area under the curve of 0.781 (p = 0.001).

The ROC curve relating angular motion to revision surgery is shown in Fig. 2, with an angular motion of 14° between the positions of flexion and extension providing the best cutoff to differentiate between the revision and BKP groups (AUC, 0.781; p = 0.001). The OR for significant between-group factors are reported in Table 3. After adjustment of the logistic regression models for age, sex, angular motion ≥14°, endplate deficit, and split type fracture, a split type fracture was the highest risk factor for revision surgery (OR, 16.5; 95% confidence interval (CI), 1.6–167.3, p = 0.018). Angular motion ≥14° increased the risk for revision 6-fold (p = 0.013), with an endplate deficit also increasing the risk for revision (OR, 5.0; 95% CI, 1.1–21.7, p = 0.032).

**Table 3:** Odds ratio (OR) of radiological findings for revision surgery after BKP.

	Crude OR	P-value	Adjusted OR*	P-value
Angular motion between flexion and extension position preop ≥14°	5.3 (1.7–17.0)	0.005	6.7 (1.5–30.4)	0.013
DISH	2.8 (0.77–10.4)	0.116		
Posterior element	1.8 (0.44–7.1)	0.421		
Pedicle fracture	1.8 (0.44–7.1)	0.421		
Endplate deficit	7.1 (2.1–24.7)	0.002	5.0 (1.1–21.7)	0.032
Posterior wall injury	3.4 (0.43–27.8)	0.245		
Split type fracture	14.0 (2.1–93.3)	0.006	16.5 (1.6–167.3)	0.018

*Adjusted for age, sex, angular motion ≥ 14°, endplate deficit and split type of fracture.

DISH, diffuse idiopathic skeletal hyperostosis; preop, pre-operatively; BKP, balloon kyphoplasty.

**Figure 3:**
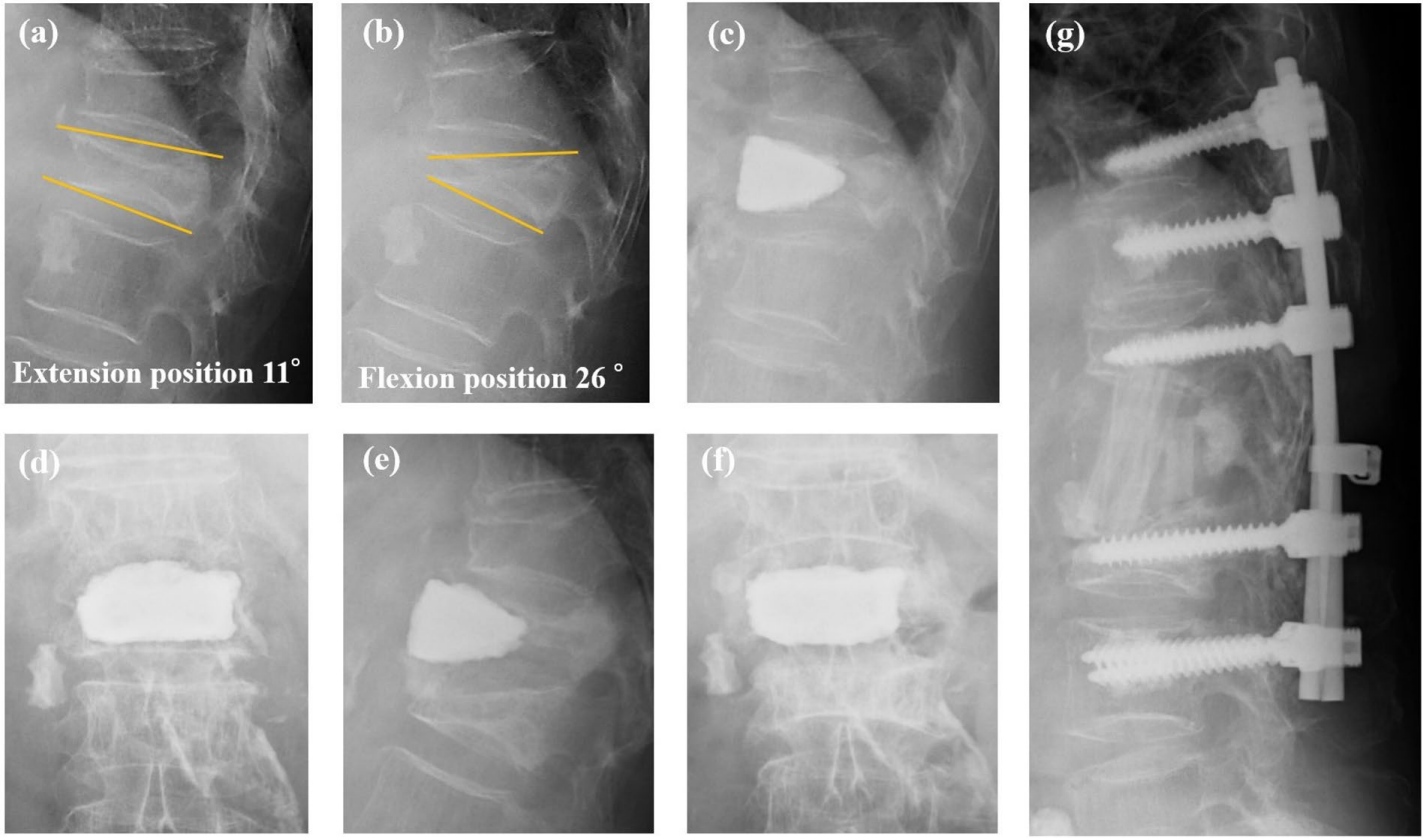
A representative case. (**a**,**b**) A 74-year-old female with an osteoporotic vertebral fracture of L1, with an large angular motion of 15° between the extension and flexion position. (**c**,**d**) Radiographs obtained at 1-week after balloon kyphoplasty. (**e**,**f**) Radiographs obtained at 2 months after balloon kyphoplasty. (**g**) L1 corpectomy and autografting, using posterior fixation from T10 to L3.

Radiographs for a representative case of revision after BKP is shown in Fig. 3. This is a 74-year-old woman who was treated by BKP for an OVF at L1. The angular motion before surgery was 15°. The patient reported a relief of her back pain after surgery, but with a subsequent exacerbation, 2 months post-surgery. Radiographs revealed cement dislodgement to the anterior and a fracture of the caudal vertebral body (L2). An L1 corpectomy was performed, with autografting and posterior fixation from T10 to L3.

## Discussion

To our knowledge, this is the first study to investigate the radiological risk factors for revision surgery after BKP. Overall, revision surgery after BKP is relatively rare, as our study shows, with an incidence rate of revision of 3.8% (4/109 BKP cases), which is only slightly higher than the rate of 1.3% reported by Yang *et al*.^8^. Our study used MRI findings associated with poor prognostic factors to select the patients for BKP^11^, which might have affected the revision rate. Considering the potential complications of revision surgery, understanding the risk factors for revision prior to the first BKP is clinically important.

Large endplate deficit and split type fractures might be associated with a higher incidence of cement leakage, increasing the risk of revision surgery. Cement leakage to an adjacent disc is frequently encountered, symptomatic neurological complications due to compression of a nerve root or the spinal cord are less frequent. However, there is increasing evidence that intradiscal leakage may lead to secondary AVFs^6,14^. The incidence of AVF is relatively high, ranging from 11% to 29% after BKP^9,15^. Based on our findings, we do not recommend BKP alone for the treatment of large endplate deficit and split type fractures. Therefore, verification of the fracture type prior to surgery is important. Kyphoplasty and short-segment pedicle screw instrumentation may be effective for these fractures^16,17^.

The greater angular motion of a fractured vertebra can be associated with greater vertebral height reduction in BKP. A significant reduction in vertebral height is a risk factor for an AVF^18^. In addition, the presence of an intravertebral cleft with angular motion is reported as a poor prognostic indicator after vertebroplasty^19^. The greater angular motion might reflect the breakage or dysfunction of the anterior spinal elements, including the anterior longitudinal ligament and annulus, which might lead to failure in maintaining the cement with the vertebral body. Anterior dislodgment of cement causes a loss of vertebral height and stability.

There is currently no universal classification for OVFs, as commonly used trauma classifications, such as the AO spine^13^ and Denis^20^ classifications, were not initially developed for osteoporotic fractures. A new classification, based on the work of the Spine Section of the German Society for Orthopaedics and Trauma, proposes 5 subgroups for osteoporotic fractures, with substantial interobserver reliability^21^. The classification includes the severity of compression and posterior wall injury. However, in our case series, severe compression and posterior wall injury were not risk factors for revision after BKP. In terms of revision surgery, no useful classification has been developed. Rather, despite posterior wall injury being a well-accepted relative contraindication for BKP, Kruger *et al*.^5^ demonstrated that BKP can be an effective low-risk procedure for geriatric patients with an OVF, with partial inclusion of the posterior wall of the vertebral body, and pain reduction was achieved immediately after surgery; consequently, patient satisfaction was very high. In our case series, there was no apparent difference in the rate of posterior wall injury between the BKP and revision groups.

Treatment of thoracolumbar fractures remains controversial. Current systematic reviews document the low level of evidence currently available to inform the treatment strategy^8,22,23^. Yang *et al*.^8^ summarized possible revision strategies for failed vertebroplasty. The surgical strategy for cement leakage into spinal canal causing neurological deficit is urgent laminectomy and fusion. Cement dislodgement or fragmentation needs anterior or posterior surgery. For infection, extensive debridement is necessary, with combined anterior and posterior surgery being the safest method for treating this kind of complication. Augmentation of pedicle screw fixation, using various bone cements (PMMA, hydroxy apatite, calcium sulfate, and calcium phosphate) is less evident, although its use as an initial procedure to improve fatigue strength of instrumentation among patients with severely osteoporosis^24^.

The limitations of our study should be acknowledged with respect to the interpretation of our findings. First, the case control design used in our study has the potential for patient selection bias, particularly since elderly patients may decline revision surgery due to existing health comorbidities. Such a selection bias could underestimate the association between severe fracture and revision surgery. Second, we did not evaluate bone status before BKP in the 11 patients who were referred to our institutions for revision surgery. However, there were no differences in terms of osteoporosis medicine, bone mineral density and postoperative therapy between the 4 patients who required revision surgery after BKP and the 105 patients who did not require revision during the prospective follow-up. Finally, the follow-up period was short. Therefore, we extended the follow-up of patients who did not receive revision surgery at 6 months after surgery. Eighty-two patients of these 105 patients were followed-up for 2 years, but none of the patients required revision surgery till the 2-year follow-up.

In conclusion, 3.8% of patients who underwent BKP for OVFs required revision surgery. A split type fracture, angular motion ≥14° and large endplate deficit (>3 mm) are risk factors for revision surgery after BKP. Treatment strategies for patients with these risk factors should be carefully evaluated, considering the inherent difficulties in performing revision surgery after BKP.
